# Supplementary material for: Clonal dominance and transplantation dynamics in hematopoietic stem cell compartments
Source: PLoS Comput Biol. 2017 Oct 9;13(10):e1005803. doi: 10.1371/journal.pcbi.1005803 (PMC5654265; doi:10.1371/journal.pcbi.1005803)
Supplement: S1 Supporting Information — Contains detailed derivations of all equations presented in the manuscript, including the details of the projection method. The analysis is carried out for unrestricted parameters, including selective effects on all parameters and permitting death in the BM space, as well as direct attachment of new daughter cells to the niche. (PDF) [file pcbi.1005803.s001.pdf]

Supporting Information (SI) for Clonal Dominance and Transplantation Dynamics in Hematopoietic Stem Cell Compartments

Peter Ashcroft[1*], Markus G. Manz[2], Sebastian Bonhoeffer[1]

1 Institut für Integrative Biologie, ETH Zürich, Universitätstrasse 16, CH-8092 Zürich, Switzerland

2 Division of Hematology, University Hospital Zürich and University of Zürich, Schmelzbergstrasse 12, CH-8091 Zürich, Switzerland

* peter.ashcroft@env.ethz.ch

Supporting Information (SI)

SI.I Model description

The full model, as depicted in Fig 1 of the main manuscript, consists of four sub-populations and six processes for each cell type (wildtype and mutant/donor). The number of host or wildtype cells located in the bone marrow (BM) is n_1, while s_1 is the number of cells of this type in the peripheral blood (PB). Likewise, n_2 and s_2 are the number of mutant/donor cells in the BM and PB, respectively. The BM has a maximum capacity of N cells, representing the finite niche space in an organism. The cell numbers are affected by birth, death, detachment from the BM, and attachment to the BM. The effect of these events and the rate at which they happen are given by the following reactions:

$$\begin{aligned}
\text{Reproduction into PB:} \quad & (n_i, s_i) \xrightarrow{[1-\rho_i(n)]\beta_i n_i} (n_i, s_i+1), && \text{(SI.1a)} \\
\text{Reproduction into BM:} \quad & (n_i, s_i) \xrightarrow{\rho_i(n)\beta_i n_i} (n_i+1, s_i), && \text{(SI.1b)} \\
\text{Death in PB:} \quad & (n_i, s_i) \xrightarrow{\delta_i s_i} (n_i, s_i-1), && \text{(SI.1c)} \\
\text{Death in BM:} \quad & (n_i, s_i) \xrightarrow{\delta_i' n_i} (n_i-1, s_i), && \text{(SI.1d)} \\
\text{Detachment:} \quad & (n_i, s_i) \xrightarrow{d_i n_i} (n_i-1, s_i+1), && \text{(SI.1e)} \\
\text{Attachment:} \quad & (n_i, s_i) \xrightarrow{a_i s_i (N-n)/N} (n_i+1, s_i-1), && \text{(SI.1f)}
\end{aligned}$$

where $n = \sum_i n_i$, and $(N-n)/N$ is the fraction of unoccupied niches. The function $\rho_i(n)$ represents the probability for the new daughter cell following a reproduction event to attach directly to the BM, rather than entering the PB. This function should satisfy $0 \leq \rho_i(n) \leq 1$, as well as $\rho_i(N) = 0$. For simplicity we choose a binary function such that

$$\rho_i(n) = \begin{cases} \varrho_i & \text{if } n < N, \\ 0 & \text{otherwise.} \end{cases} \qquad \text{(SI.2)}$$

We express the death rate in the BM, δ_i', in terms of the original death rate δ_i, such that $\delta_i' = \alpha\delta_i$. With this parametrisation, setting $\alpha = 0$ prevents death from occurring within the BM,

and $\alpha = 1$ makes HSC death independent of the environment. As the BM is a more favourable environment for the HSCs, we expect $0 \leq \alpha \leq 1$. As α is effectively a property of the environment, we assume it is identical for both host and mutant/donor cells. We note here that there are no direct interaction terms between host cells and mutant/donor cells (no switching from type 1 to type 2 etc.). We use the following parametrisation for the reaction parameters:

$$\beta_1 = \beta, \qquad \beta_2 = (1 + \varepsilon\gamma_\beta)\beta, \tag{SI.3a}$$
$$\delta_1 = \delta, \qquad \delta_2 = (1 + \varepsilon\gamma_\delta)\delta, \tag{SI.3b}$$
$$\delta'_1 = \alpha\delta_1 \qquad \delta'_2 = \alpha\delta_2, \tag{SI.3c}$$
$$d_1 = d, \qquad d_2 = (1 + \varepsilon\gamma_d)d, \tag{SI.3d}$$
$$a_1 = a, \qquad a_2 = (1 + \varepsilon\gamma_a)a, \tag{SI.3e}$$
$$\varrho_1 = \varrho \qquad \varrho_2 = (1 + \varepsilon\gamma_\varrho)\varrho. \tag{SI.3f}$$

Here ε represents the strength of selection. For analysis purposes discussed below, we assume $0 \leq \varepsilon \ll 1$. For $\varepsilon = 0$, we have the so-called neutral model. The parameters γ_j ($j \in \{\beta, \delta, d, a, \varrho\}$) satisfy $\gamma_j = \mathcal{O}(1)$. They allow the parameters to be varied independently. In the main manuscript we restrict these parameters to $\gamma_\beta = 1$ and $\gamma_\delta = \gamma_d = \gamma_a = \gamma_\varrho = 0$, along with $\alpha = \varrho = 0$, but we carry out the analysis in general here.

Considering a steady-state system in the absence of the mutant/donor cells ($n_2 = s_2 = 0$), the deterministic dynamics of the system are described by ordinary differential equations (ODEs):

$$\frac{\mathrm{d}n_1}{\mathrm{d}t} = (\varrho\beta - d - \alpha\delta)n_1 + as_1\frac{N - n_1}{N}, \tag{SI.4a}$$
$$\frac{\mathrm{d}s_1}{\mathrm{d}t} = [d + (1 - \varrho)\beta]n_1 - \left(\delta + a\frac{N - n_1}{N}\right)s_1. \tag{SI.4b}$$

From these ODEs we can obtain expressions for the equilibrium size of the BM and PB compartments, n^* and s^*, respectively. These are given by

$$n^* = N\left(1 - \frac{\delta(d + \alpha\delta - \varrho\beta)}{a(\beta - \alpha\delta)}\right), \tag{SI.5a}$$
$$s^* = \frac{(\beta - \alpha\delta)}{\delta}n^*. \tag{SI.5b}$$

Table SI.1. Deduced parameter value ranges (units are per day). Here we have fixed $\ell = 3$ minutes and $\varrho = 0$.

Parameter	Expression		Value (per day)							
		s^*:	1 cell				100 cells			
		α:	0	10^{-4}	10^{-2}	1	0	10^{-4}	10^{-2}	1
δ	$\frac{\beta n^*}{s^*+\alpha n^*}$		250	130	2.5	0.026	— 2.5 —		1.3	0.025
d	$\frac{s^*}{\ell n^*} - \beta$		——— 0.022 ———				——— 4.8 ———			
a	$\left(\frac{1}{\ell} - \frac{\beta n^*}{s^*+\alpha n^*}\right)\frac{N}{N-n^*}$		23,000	35,000	— 48,000 —		——— 48,000 ———			

From Eq (SI.5b), the death rate δ can be expressed in terms of β, s^*, n^*, and the variable parameter α. Furthermore, a cell in the PB compartment (in equilibrium) can either die with rate δ or attach to an unoccupied niche with rate $a(N - n^*)/N$. Hence the expected lifetime of a cell in the PB is $\ell = [\delta + a(N - n^*)/N]^{-1}$. From this we can obtain and expression for the attachment rate a. Finally, d is found from Eq (SI.4a). Thus for the non-valued parameters δ, d, and a, we have

$$\delta = \frac{\beta n^*}{s^* + \alpha n^*}, \tag{SI.6a}$$

$$d = \frac{s^*}{\ell n^*} - (1 - \varrho)\beta, \tag{SI.6b}$$

$$a = \left(\frac{1}{\ell} - \frac{\beta n^*}{s^* + \alpha n^*}\right)\frac{N}{N - n^*} \tag{SI.6c}$$

These expressions, and the possible range of values, are given in Table 2 of the main manuscript for $\alpha = \varrho = 0$. A further example is provided in Table SI.1 of this document for $\alpha \neq 0$. By introducing death in the niche, we affect the balance of cells leaving and entering each compartment. The total number of cells produced (βn^*) must be matched by the total number of cells that die. As we have increased the number of cells that are susceptible to death, we must decrease the death rate δ. Hence, δ is a decreasing function of α. To replace the cells that die within the BM, we need to increase the flux of cells from the PB to the BM. In other words, death in the BM must be compensated by an increased rate of migration between the PB and BM compartments. Hence, we have that a is an increasing function of α. The detachment rate d is independent of α, but is an increasing function of ϱ to ensure enough cells migrate to the PB.

For completeness, the deterministic dynamics of the two-species system are described by the

ODEs:

$$\frac{\mathrm{d}n_1}{\mathrm{d}t} = (\varrho\beta - d - \alpha\delta)n_1 + as_1\frac{N-n}{N}, \tag{SI.7a}$$
$$\frac{\mathrm{d}n_2}{\mathrm{d}t} = (\varrho_2\beta_2 - d_2 - \alpha\delta_2)n_2 + a_2s_2\frac{N-n}{N}, \tag{SI.7b}$$
$$\frac{\mathrm{d}s_1}{\mathrm{d}t} = [d + (1-\varrho)\beta]n_1 - \left(\delta + a\frac{N-n}{N}\right)s_1, \tag{SI.7c}$$
$$\frac{\mathrm{d}s_2}{\mathrm{d}t} = [d_2 + (1-\varrho_2)\beta_2]n_2 - \left(\delta_2 + a_2\frac{N-n}{N}\right)s_2, \tag{SI.7d}$$

with $n = n_1 + n_2$.

SI.II Initial dynamics and chimerism

In the scenario of donor cell transplantation, the PB compartment initially contains more cells than the equilibrium value in the neutral model ($s_1 + s_2 > s^*$). This leads to a net flux of cells attaching to the BM, and hence $n_1 + n_2 > n^*$. The continuing attachment–detachment dynamics allows the donor cells to replace the host cells in the BM. Meanwhile, surplus cells in the PB are dying off and the population relaxes to its equilibrium size ($n_1 + n_2 = n^*$ and $s_1 + s_2 = s^*$). The host cells in the BM are effectively displaced by the new donor HSCs. Once the equilibrium is reached the initial dynamics end. We find that the effect of selection, ε, only acts on a long timescale, and has little influence on the outcome of initial dynamics. Therefore we treat the donor cells as neutral until the long-term noise-driven dynamics (discussed below) take over.

For small doses of donor HSCs, and especially the case of $\mathcal{S} = 1$ when a *de novo* mutant is generated, the number of additional cells in the BM ($n_1 + n_2 - n^*$) is small. We can neglect this expansion of the BM pool and approximate the number of occupied niches as a constant, n^*. By considering only first-order reactions of the donor HSCs (i.e. the injected cells either die or attach to the BM, there is no reproduction or detachment), we can predict the number of donor HSCs that attach to the BM. We find

$$n_2 = \frac{a(N-n^*)/N}{\delta + a(N-n^*)/N}\mathcal{S} = \left(1 - \frac{\ell\beta n^*}{s^* + \alpha n^*}\right)\mathcal{S}. \tag{SI.8}$$

Hence for small doses the chimerism achieved is directly proportional to the dose size. The

simplified process that led to Eq (SI.8) (only first-order dynamics) predicts $s_2 = 0$ after the initial dynamics, which is incorrect. However, we know the relation between s_i and n_i in equilibrium [Eq (SI.5b)], which tells us that $s_2 = (\beta - \alpha\delta)n_2/\delta$.

If the dose of donor HSCs is large enough, all niches become occupied and the BM compartment is saturated. In this case a niche that has been vacated (either by detachment or death) is immediately filled, either by a cell from the PB or from a reproduction event. For example, if an n_1 cell detaches from the BM or dies within the BM, then it can be immediately replaced by an s_2 cell from the PB, or by an n_2 cell following reproduction with attachment. Here we consider these combined detachment–attachment dynamics. As the vacant niche is immediately occupied, we approximate the rate of the coupled detachment–attachment reaction as a single exponential step determined by the rate at which the niche becomes available (either d or $\alpha\delta$).

For s_1 – the number of wildtype cells in the PB – we have an increase due to reproduction with rate βn_1 (niches are saturated so the new daughter cell enters the PB with certainty), and a decrease due to death at rate δs_1. Furthermore, s_1 will increase if an s_2 attaches to the BM following the detachment of an n_1 cell; this process occurs at rate $dn_1 s_2/s$, where $s = s_1 + s_2$ and s_2/s is the probability that an s_2 cell attaches rather than s_1. Also, s_1 can decrease if it attaches to a niche vacated by n_2 cell. Finally, an s_1 cell can attach to a niche that opens due to the death of the occupant, which happens with rate $\alpha\delta N s_1/s$ (here we assume $n_1 + n_2 = N$). Similar dynamics follow for s_2. Hence, for the PB cells we have the following simplified equations

$$\frac{\mathrm{d}s_1}{\mathrm{d}t} = \beta n_1 - \delta s_1 + d\left(n_1\frac{s_2}{s} - n_2\frac{s_1}{s}\right) - \alpha\delta N\frac{s_1}{s(t)}, \tag{SI.9a}$$

$$\frac{\mathrm{d}s_2}{\mathrm{d}t} = \beta n_2 - \delta s_2 + d\left(n_2\frac{s_1}{s} - n_1\frac{s_2}{s}\right) - \alpha\delta N\frac{s_2}{s(t)}. \tag{SI.9b}$$

The size of the PB pool follows the linear equation and solution

$$\begin{aligned} \frac{\mathrm{d}s}{\mathrm{d}t} &= (\beta - \alpha\delta)N - \delta s \\ \Rightarrow \quad s(t) &= \left(s(0) - \frac{(\beta - \alpha\delta)N}{\delta}\right)e^{-\delta t} + \frac{(\beta - \alpha\delta)N}{\delta}. \end{aligned} \tag{SI.10}$$

Here we assume the $N - n^*$ unoccupied niches are immediately filled after injection, such that

$s(0) = s^* + \mathcal{S} - (N - n^*)$ and $n(t \geq 0) = N$.

For the cells in the BM, we construct the coupled dynamics in a similar way; a niche is cleared at rate $(d + \alpha\delta)N$, and the cell is replaced immediately by one from the PB, or by reproduction within the niche. Wildtype BM cells can increase following the detachment/death of an n_2 cell with rate $(d+\alpha\delta)n_2[(s_1/s) + (n_1/N)]$. Here s_1/s represents the attachment of an s_1 cell (as opposed to s_2), and n_1/N the reproduction within the niche of an n_1 cell. The decrease of n_1 follows analogously, and occurs with rate $(d + \alpha\delta)n_1[(s_2/s) + (n_2/N)]$. Hence, we can now write down the approximate ODEs for this scenario:

$$\frac{\mathrm{d}n_1}{\mathrm{d}t} = (d + \alpha\delta)\left(n_2\frac{s_1}{s} - n_1\frac{s_2}{s}\right), \tag{SI.11a}$$
$$\frac{\mathrm{d}n_2}{\mathrm{d}t} = (d + \alpha\delta)\left(n_1\frac{s_2}{s} - n_2\frac{s_1}{s}\right), \tag{SI.11b}$$

where the terms due to reproduction have cancelled out. Although not obvious from above, Eq (SI.9) and Eq (SI.11) are actually linear in the n_i and s_i. Writing $n_1 = N - n_2$ and $s_1 = s - s_2$ we have

$$\frac{\mathrm{d}n_2}{\mathrm{d}t} = -(d + \alpha\delta)n_2 + \frac{(d+\alpha\delta)N}{s}s_2, \tag{SI.12a}$$
$$\frac{\mathrm{d}s_2}{\mathrm{d}t} = (\beta + d)n_2 - \left(\delta + \frac{(d+\alpha\delta)N}{s}\right)s_2, \tag{SI.12b}$$

along with $n_2(0) = N - n^*$ and $s_2(0) = \mathcal{S} - (N - n^*)$. Using these equations we can predict the value of n_2 once the PB has returned to its equilibrium size.

SI.III Clonal dominance

SI.III.1 General approach and the neutral scenario

Once the mutant/donor cells have established themselves within the BM compartment, we want to know if and how quickly this clone expands. To this end we use the projection method of Constable *et al.* [41, 42]. This analysis is more intuitive when the mutant/donor cells have no selective advantage, so we first discuss the neutral scenario. In this case once the BM and PB compartments return to their equilibrium size ($n_1 + n_2 = n^*$ and $s_1 + s_2 = s^*$) there is no

deterministic dynamics; as the mutant/donor cells are neutral when compared to the host, we have effectively returned to a healthy and stable host. However, the stochastic dynamics of the individual-based model continue. Cells are continually migrating between the BM and PB compartments, and reproduction and death events go on. If cell numbers increase [decrease] then the flux of cells leaving the system increases [decreases] until the equilibrium is restored. Therefore the deterministic dynamics constrains cell numbers to $n_1 + n_2 = n^*$ and $s_1 + s_2 = s^*$. Cell number fluctuations, however, change the balance between host and mutant/donor cells over time, and we observe diffusion along the equilibrium line. Eventually, this diffusion leads to the extinction of either the host or the mutant/donor population of HSCs.

We first move from the master equation – the exact probabilistic description of the stochastic dynamics – to a set of stochastic differential equations (SDEs) [43]. To this end we introduce the variables $\mathbf{x} = (s_1, s_2, n_1, n_2)^{\mathrm{T}}/N$ and expand the master equation in powers of $1/N$, using the fact that N is a large parameter. The evolution of $\mathbf{x}$ is determined by the set of SDEs

$$\frac{\mathrm{d}x_i}{\mathrm{d}t} = A_i(\mathbf{x}) + \frac{1}{\sqrt{N}}\eta_i(t), \tag{SI.13}$$

where the A_i are the drift terms representing the deterministic dynamics, and the η_i are Gaussian noise terms which describe the diffusion. The η_i have zero expectation value and correlator

$$\langle \eta_i(t)\eta_j(t')\rangle = \delta(t-t')B_{ij}(\mathbf{x}). \tag{SI.14}$$

Here $\langle\cdot\rangle$ represents the expectation value over many realisations of the noise. The exact forms of

the drift vector $\mathbf{A}$ and diffusion matrix $\mathbf{B}$ (valid for $n < N$) are

$$\mathbf{A}(\mathbf{x}) = \begin{pmatrix} [d + (1-\varrho)\beta]x_3 - (\delta + ay)x_1 \\ [d + (1-\varrho)\beta]x_4 - (\delta + ay)x_2 \\ (\varrho\beta - d - \alpha\delta)x_3 + ayx_1 \\ (\varrho\beta - d - \alpha\delta)x_4 + ayx_2 \end{pmatrix}, \tag{SI.15a}$$

$$\mathbf{B}(\mathbf{x}) = \begin{pmatrix} [d + (1-\varrho)\beta]x_3 + (\delta + ay)x_1 & 0 & -dx_3 - ayx_1 & 0 \\ 0 & [d + (1-\varrho)\beta]x_4 + (\delta + ay)x_2 & 0 & -dx_4 - ayx_2 \\ -dx_3 - ayx_1 & 0 & (\varrho\beta + d + \alpha\delta)x_3 + ayx_1 & 0 \\ 0 & -dx_4 - ayx_2 & 0 & (\varrho\beta + d + \alpha\delta)x_4 + ayx_2 \end{pmatrix}. \tag{SI.15b}$$

where we have used the shorthand $y = 1 - x_3 - x_4$, which is the fraction of unoccupied niches. Eq (SI.13) is an approximate description of the full stochastic dynamics. If we neglect the noise term, we recover the ODEs Eq (SI.7).

The set of points at which the drift vector $\mathbf{A}$ is zero is known in dynamical systems theory as the slow manifold. Our slow manifold, $\mathbf{x}^*$, satisfies the conditions $x_1^* + x_2^* = s^*/N$ and $x_3^* + x_4^* = n^*/N$, as well as $x_1^* = [(\beta - \alpha\delta)/\delta]x_3^*$ and $x_2^* = [(\beta - \alpha\delta)/\delta]x_4^*$. The first two conditions describe the equilibrium size of the PB and BM compartments, and the latter conditions describe the balance between reproduction and cell death. These four conditions can be satisfied parametrically by

$$\mathbf{x}^*(z) = \left(\frac{\beta - \alpha\delta}{\delta}(\xi - z),\ \frac{\beta - \alpha\delta}{\delta} z,\ \xi - z,\ z \right)^{\mathrm{T}}, \tag{SI.16}$$

where $\xi = n^*/N$ and $z \in [0, \xi]$. Hence, our slow manifold is a line through the 4-dimensional state-space. In other words, if we were to measure the number of donor cells in the BM then we could infer the number of host cells from the system-size constraint. These numbers, along with knowledge of the reproduction and death rates, can be used to infer cell numbers in the PB. Therefore we only need to keep track of one variable, z, to describe our system.

As there is no deterministic drift along our slow manifold (in the neutral scenario), the

time-evolution of the parametric coordinate z satisfies

$$\frac{\mathrm{d}z}{\mathrm{d}t} = \frac{1}{\sqrt{N}}\eta(t), \tag{SI.17}$$

where $\eta(t)$ is Gaussian noise with zero expectation value and correlator

$$\langle \eta(t)\eta(t')\rangle = \delta(t-t')\widetilde{B}_{11}(z). \tag{SI.18}$$

The expansion (or contraction) of the mutant/donor clone is completely specified by this noise correlator $\widetilde{B}_{11}$, but it is not yet determined. To find it we must project the approximate dynamics [Eq (SI.13)] onto our slow manifold $\mathbf{x}^*$ [41, 42]. In this way we capture the effects of the cell number fluctuations described above. To achieve this we note that the Jacobian matrix of the drift vector along the slow manifold, $\mathbf{A}(\mathbf{x}^*)$, has a zero eigenvalue. This corresponds to the direction in which there is no deterministic motion, and hence the associated eigenvector is directed along the slow manifold. Thus we use the eigenvectors of $\mathbf{A}(\mathbf{x}^*)$ as a basis, onto which we decompose the SDEs Eq (SI.13). Selecting only the component along the slow manifold, we find the correlator

$$\widetilde{B}_{11}(z) = 2\mathcal{B}\, z(\xi - z), \tag{SI.19}$$

where the constant $\mathcal{B}$ is given by

$$\begin{aligned}\mathcal{B} &= \frac{\beta[d+(1-\varrho)\beta][d+\alpha\delta(1-\varrho)]\delta^2}{\xi\{\beta\delta+\beta d+d\delta+\alpha\delta[\beta-(d+\alpha\delta)]-\varrho\beta[\beta+\delta-\alpha\delta]\}^2}\\ &= \frac{\beta n^* N(s^*+\alpha n^*)[s^*+\alpha n^*-n^*\ell(1-\varrho)\beta]}{[(n^*+s^*)(s^*+\alpha n^*)-n^*s^*\ell\beta]^2}.\end{aligned} \tag{SI.20}$$

The first line of this equation expresses the diffusion constant in terms of the reaction parameters, while the second line uses Eq (SI.6) to express $\mathcal{B}$ in terms of the experimentally-observed parameters, as well as α and ϱ.

By rescaling time and the coordinate z in Eq (SI.19), it can be shown that the dynamics along the slow manifold are equivalent to those of the Moran model [41, 42].

We can use the standard results of Brownian motion to determine the probability that the mutant/donor clone expands to a given fraction σ, and the mean time for this to happen [43].

For example, $\sigma = 0.5$ corresponds to a clone that represents 50% of all HSCs. We assume the dynamics starts at a point $\mathbf{x}^*(z_0)$ along the slow manifold. Here $z_0 = n_2/N$, where n_2 is the number of mutant/donor cells that make up the BM compartment at the end of the initial dynamics as described in the previous section. In particular, for the case of disease spread ($\mathcal{S} = 1$), z_0 can be found explicitly from Eq (SI.8). The probability that the mutant/donor HSCs reach a fraction $\sigma \leq 1$ is given by

$$\phi(z_0, \sigma) = \frac{z_0}{\sigma\xi}. \tag{SI.21}$$

The mean time for this expansion (i.e. the mean conditional time) is given by

$$T_\xi(z_0, \sigma) = \frac{N}{\mathcal{B}}\left[\frac{\xi - z_0}{z_0}\log\left(\frac{\xi}{\xi - z_0}\right) + \frac{1-\sigma}{\sigma}\log(1-\sigma)\right]. \tag{SI.22}$$

For $\sigma = 1$, we recover the fixation probability and mean conditional fixation time of the mutant/donor cells.

SI.III.2 With selection

We now repeat this analysis for the non-neutral case, i.e. the mutant/donor cells have a selective (dis)advantage. For $\varepsilon > 0$, the drift vector in Eq (SI.13) becomes

$$\mathbf{A}^{(\varepsilon)}(\mathbf{x}) = \begin{pmatrix} [d + (1-\varrho)\beta]x_3 - (\delta + ay)x_1 \\ [d + (1-\varrho)\beta]x_4 - (\delta + ay)x_2 \\ (\varrho\beta - d - \alpha\delta)x_3 + ayx_1 \\ (\varrho\beta - d - \alpha\delta)x_4 + ayx_2 \end{pmatrix} + \varepsilon \begin{pmatrix} 0 \\ [d\gamma_d + (1-\varrho)\beta\gamma_\beta - \varrho\beta\gamma_\varrho]x_4 - (\delta\gamma_\delta + a\gamma_a y)x_2 \\ 0 \\ [\varrho\beta(\gamma_\varrho + \gamma_\beta) - d\gamma_d - \alpha\delta\gamma_\delta]x_4 + a\gamma_a yx_2 \end{pmatrix} + \mathcal{O}(\varepsilon^2). \tag{SI.23}$$

We assume there is no change in the noise correlator as terms $\mathcal{O}(\varepsilon/N)$ are negligible; hence we have $\mathbf{B}^{(\varepsilon)}(\mathbf{x}) \approx \mathbf{B}^{(0)}(\mathbf{x}) = \mathbf{B}(\mathbf{x})$, as given in Eq (SI.15b).

As there is always some deterministic drift due to the effect of selection, by definition there is no slow manifold. However, the number of cells leaving the system will still be balanced by production as described above. This balance point describes a subspace around which the cell numbers fluctuate. With $\varepsilon > 0$ there will be a tendency for the advantageous cells to replace their counterparts. This induces a slow drift along the subspace. By integrating the

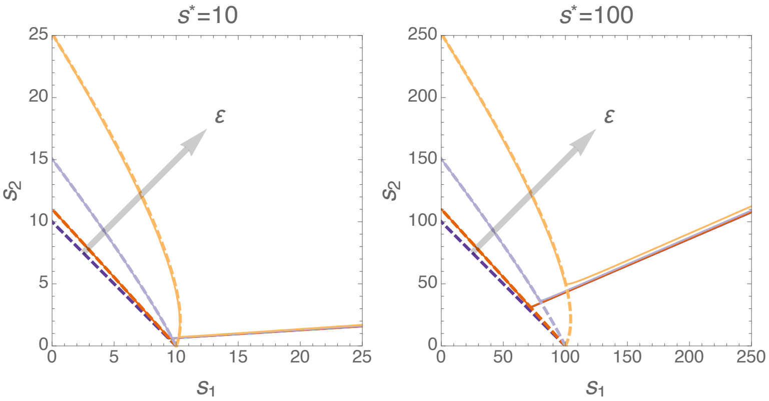

Fig SI.1. Time course of ODEs Eq (SI.7) (solid lines) with an initial dose of $\mathcal{S} = 5,000$ donor cells into a steady-state host. Selection strengths in this figure are $\varepsilon \in \{0, 0.1, 0.5, 1.5\}$ (increasing in direction of arrow). Dashed lines are the approximate slow subspaces, $\widetilde{\mathbf{x}}$, recovered from the projection method. For $\varepsilon = 0$ the ODE time course stops once it reaches the slow manifold. Increasing ε moves the slow subspace away from the $\varepsilon = 0$ slow manifold. Here we have selection acting only on the reproduction rate ($\gamma_\beta = 1$, $\gamma_a = \gamma_\delta = \gamma_d = 0$), and we have $\ell = 3$ minutes. In the left panel $s^* = 10$ and in the right panel $s^* = 100$. We here set $\alpha = \varrho = 0$. Remaining parameters are as in Table 1 of the main manuscript.

ODEs Eq (SI.7) for a long time we can visualise this subspace. Examples are shown in Fig SI.1 for different selection strengths. In the absence of selection ($\varepsilon = 0$), the trajectory from the ODEs Eq (SI.7) stops once the slow manifold has been reached. This is the reason why the dashed line is not accompanied by a solid trajectory for $\varepsilon = 0$. For $\varepsilon > 0$, the advantageous mutant/donor cells are able to maintain a higher equilibrium population size than the host. Hence the slow subspaces always lie above the neutral slow manifold.

We can use the projection method to calculate the approximate form of the slow subspace, $\widetilde{\mathbf{x}}(z)$ [41, 42]. This takes the form $\widetilde{\mathbf{x}}(z) = \mathbf{x}^*(z) + \varepsilon f(z)$, and again we only need one variable to describe our system. These approximations are shown as dashed lines in Fig SI.1, and they remain highly accurate (compared to numerical integration of the ODEs Eq (SI.7)) even for large values of ε.

Using the same eigenbasis from the neutral model we can project the dynamics onto the slow

subspace [41, 42]. The SDE describing the motion along the slow subspace is

$$\begin{aligned}\frac{\mathrm{d}z}{\mathrm{d}t} &= \widetilde{A}_1(z) + \frac{1}{\sqrt{N}}\eta(t),\\ \langle \eta(t)\rangle &= 0,\\ \langle \eta(t)\eta(t')\rangle &= \delta(t-t')\widetilde{B}_{11}(z).\end{aligned} \tag{SI.24}$$

The drift along this subspace, $\widetilde{A}_1(z)$, is of the form

$$\widetilde{A}_1(z) = \varepsilon \mathcal{A}\, z(\xi - z), \tag{SI.25}$$

where the constant $\mathcal{A}$ is given by

$$\mathcal{A} = \frac{\delta}{\xi}\frac{\gamma_a(\beta-\alpha\delta)(d+\alpha\delta-\varrho\beta) - \gamma_d d(\beta-\alpha\delta) + \gamma_\beta \beta[d+\alpha\delta(1-\varrho)] - \gamma_\delta[\alpha\delta(\beta-\alpha\delta) - \beta(\varrho\beta - d - \alpha\delta)] + \gamma_\varrho \varrho\beta(\beta-\alpha\delta)]}{\beta\delta + \beta d + d\delta + \alpha\delta(\beta - d - \alpha\delta) - \varrho\beta[\beta + (1-\alpha)\delta]}. \tag{SI.26}$$

By setting $\alpha = \varrho = 0$, we recover

$$\mathcal{A} = \frac{d\beta\delta}{\xi(d\beta + d\delta + \beta\delta)}(\gamma_\beta + \gamma_a - \gamma_\delta - \gamma_d). \tag{SI.27}$$

This expression gives an important result; it doesn't matter in which sense the cells are advantageous. A cell with an increased reproduction rate ($\gamma_\beta = 1$; $\gamma_a = \gamma_\delta = \gamma_d = 0$) has the same advantage as a cell which attaches to the BM more quickly ($\gamma_a = 1$; $\gamma_\beta = \gamma_\delta = \gamma_d = 0$). All that matters is the cumulative advantage, $\gamma_\beta + \gamma_a - \gamma_\delta - \gamma_d$. For this reason we only consider a reproductive advantage in the presented results in the main manuscript.

Again using the standard results of Brownian motion [43], we find the probability for the donor cells to represent a fraction σ of the population to be

$$\phi(z_0, \sigma) = \frac{1 - e^{-\Lambda z_0}}{1 - e^{-\Lambda\sigma\xi}}, \qquad \text{with} \quad \Lambda = \frac{\varepsilon N \mathcal{A}}{\mathcal{B}}. \tag{SI.28}$$

This solution is of the same form as that obtained for a Moran model [41, 42]. Although a closed-form solution is possible for the mean conditional time to reach size σ, it is too long to

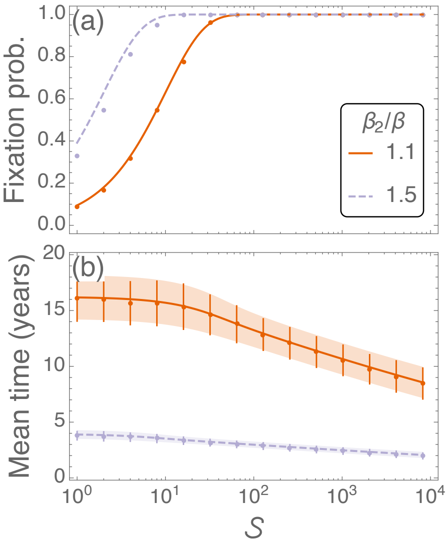

Fig SI.2. Fixation probability (a) and time (b) of donor cells in a non-preconditioned host. On the horizontal axis we plot the initial dose of donor HSCs, $\mathcal{S} \in \{1, 2, 4, 8, \ldots, 8192\}$. Symbols are results from 10^3 simulations of the stochastic model (with associated standard deviations). Lines are predictions from Eq (SI.28) and Eq (SI.29). The shaded region in (b) is the standard deviation calculated from the second moment, Eq (SI.30). Here we have $s^* = 100$ cells, and $\ell = 3$ minutes, along with $\alpha = \varrho = 0$. Remaining parameters are as in Table 1 of the main manuscript.

display here. Instead we use an algebraic software package to solve the second-order differential equation

$$T_\xi(z_0, \sigma) = \frac{\theta(z_0, \sigma)}{\phi(z_0, \sigma)}, \qquad \frac{\partial^2 \theta(z_0, \sigma)}{\partial z_0^2} + \Lambda \frac{\partial \theta(z_0, \sigma)}{\partial z_0} = -\frac{N}{\mathcal{B}} \frac{\phi(z_0, \sigma)}{z_0(\xi - z_0)}, \qquad \theta(0) = \theta(\sigma\xi) = 0. \tag{SI.29}$$

Example results from Eq (SI.28) and Eq (SI.29) are shown in Fig SI.2.

Furthermore, we can write down a set of equations for the second moment of the conditional time for the mutant/donor clone to reach size σ [44]. The second moment, $\langle T_\xi^2(z_0, \sigma)\rangle$, is dependent on the first moment of the conditional time, and hence we must solve the coupled equations:

$$\frac{\partial^2 \theta_1(z_0, \sigma)}{\partial z_0^2} + \Lambda \frac{\partial \theta_1(z_0, \sigma)}{\partial z_0} = -\frac{N}{\mathcal{B}} \frac{\phi(z_0, \sigma)}{z_0(\xi - z_0)}, \qquad \theta_1(0) = \theta_1(\sigma\xi) = 0, \tag{SI.30a}$$

$$\frac{\partial^2 \theta_2(z_0, \sigma)}{\partial z_0^2} + \Lambda \frac{\partial \theta_2(z_0, \sigma)}{\partial z_0} = -2\frac{N}{\mathcal{B}} \frac{\theta_1(z_0, \sigma)}{z_0(\xi - z_0)}, \qquad \theta_2(0) = \theta_2(\sigma\xi) = 0, \tag{SI.30b}$$

$$\langle T_\xi^2(z_0, \sigma)\rangle = \frac{\theta_2(z_0, \sigma)}{\phi(z_0, \sigma)}, \tag{SI.30c}$$

where the first equation is for the first moment [identical to Eq (SI.29)], and the second equation is for the second moment. The predicted deviation calculated from this second moment is shown in Fig SI.2(b).

Full details of all these calculations are found in the accompanying Mathematica notebook file, which can be found at `https://github.com/ashcroftp/clonal-hematopoiesis-2017`.

SI.III.3 Further neutral-model calculations

The expansion of neutral clones deserves some further attention, as this process could provide valuable insight into human hematopoiesis. Firstly, we look at the case $\alpha = \varrho = 0$ and ask how does the mean conditional time to σ-level clonality vary depending on the choice of model parameters. Using Eq (SI.8), along with $\mathcal{S} = 1$, we have an expression for our initial level of clonality:

$$z_0 = \frac{1}{N}\frac{s^* - n^*\beta\ell}{s^*}. \tag{SI.31}$$

From Eq (SI.20), we also have the diffusion constant

$$\mathcal{B} = \frac{\beta N}{s^*}\frac{\frac{s^*}{n^*} - \beta\ell}{\left(1 + \frac{s^*}{n^*} - \beta\ell\right)^2}. \tag{SI.32}$$

Therefore, our mean time [Eq (SI.22)] satisfies

$$\langle T_\xi(z_0,\sigma)\rangle = \frac{s^*}{\beta}\frac{\left(1 + \frac{s^*}{n^*} - \beta\ell\right)^2}{\frac{s^*}{n^*} - \beta\ell}\left[\left(\frac{n^* s^*}{s^* - n^*\beta\ell} - 1\right)\log\left(\frac{1}{1 - \frac{s^* - n^*\beta\ell}{n^* s^*}}\right) + \frac{1-\sigma}{\sigma}\log(1-\sigma)\right]. \tag{SI.33}$$

Now assuming that terms $\mathcal{O}(N)$ are much larger than terms $\mathcal{O}(1)$, that $s^*/n^* \ll 1$ (blood compartment much smaller than bone marrow in equilibrium) and $\beta\ell \ll 1$ (migration dynamics are faster than reproduction), and that $\sigma \ll 1$, we can approximate our mean time as

$$\langle T_\xi(z_0,\sigma)\rangle \approx \frac{1}{\beta}\frac{\sigma}{2}\frac{n^* s^*}{s^* - n^*\beta\ell}. \tag{SI.34}$$

Note that we are also constrained to $s^* - n^*\beta\ell > 0$, which comes from the fact that our attachment and detachment parameters a and d must be positive. If $s^* \gg n^*\beta\ell$, then we have

$\langle T_\xi(z_0,\sigma)\rangle \approx \sigma n^*/(2\beta)$, which is independent of ℓ and s^*. As expected, the reproduction rate β is the dominant parameter determining the time to clonality.

When considering the case of $\alpha \neq 0$ and $\rho \neq 0$, we turn to a graphical representation to highlight the parameter dependence. These results are shown in supplementary figures S1 and S2. We find that allowing equal death in both compartments ($\alpha = 1$) or daughter cells to enter the niche directly ($\varrho = 1$) has little to no effect on the time to clonality.

Finally, we can obtain a closed-form solution of the second moment equation [Eq (SI.30)] in the absence of selection. We find

$$\langle T_\xi^2(z_0,\sigma)\rangle = \frac{2N}{\mathcal{B}}\left[\langle T_\xi(z_0,\sigma)\rangle\left(\frac{1-\sigma}{\sigma}\log(1-\sigma)-1\right)+\frac{N}{\mathcal{B}}\left(\mathrm{Li}_2(\sigma)-\mathrm{Li}_2\left(\frac{z_0}{\xi}\right)\right)\right], \quad \text{(SI.35)}$$

where $\mathrm{Li}_2(z)$ is the second-order polylogarithmic function.

SI.IV Engraftment into a preconditioned host

Even if the BM compartment is empty, in the stochastic model there is a finite probability that all donor cells die before they engraft. We write $\psi = \Pr(n+s=0,\, t\to\infty)$ for the extinction probability of a single cell. Therefore, the probability that a single donor HSC will reconstitute the preconditioned host is $\varphi = 1-\psi = \Pr(n+s>0,\, t\to\infty)$. For doses of size $\mathcal{S}$, the reconstitution probability is $\varphi = 1-\psi^{\mathcal{S}}$. For a first-approximation of this probability, we assume that the donor cells can only either attach to the BM niches or die. The probability that a single HSC in the PB compartment dies is $\psi = \delta/(\delta+a)$. Thus the approximate reconstitution probability is

$$\varphi = 1-\left(\frac{\delta}{\delta+a}\right)^{\mathcal{S}}. \quad \text{(SI.36)}$$

Here we have assumed that all niches are unoccupied, such that the attachment rate per cell is $a(N-0)/N = a$.

We can also consider multiple attachments and detachments, which could occur before a cell establishes a sustainable population or dies. As above, the probability that a single HSC in the PB compartment dies is $\delta/(\delta+a)$. Alternatively, the HSC can attach to the niche with probability $a/(\delta+a)$. The probability that the cell then detaches from the niche is $d/(d+\beta+\alpha\delta)$,

the probability of dying within the niche is $\alpha\delta/(d+\beta+\alpha\delta)$, and the probability of reproducing is $\beta/(d+\beta+\alpha\delta)$. Again we have assumed that the number of occupied niches is negligible. Under these processes, the probability that a single donor HSC becomes extinct is given by

$$\psi = \frac{\delta}{\delta + a} + \frac{a}{\delta + a} f(\psi), \tag{SI.37}$$

where $f(\psi)$ represents the processes that occur once the cell has entered the BM compartment. It is given by

$$f(\psi) = \frac{\alpha\delta}{d+\beta+\alpha\delta} + \frac{d}{d+\beta+\alpha\delta}\psi + \frac{\beta}{d+\beta+\alpha\delta}\left[(1-\varrho)\psi f(\psi) + \varrho f^2(\psi)\right]. \tag{SI.38}$$

Here the first term is the death of the cell within the BM compartment. The second term represents detachment and the cell is back where it started, so this is multiplied by ψ. The third term represents reproduction: either one offspring is ejected to the PB (hence ψ) and the other remains in the niche $[f(\psi)]$, or both offspring remain in the BM $[f^2(\psi)]$. Solving Eq (SI.38) for $f(\psi)$, and then using this to solve Eq (SI.37), we find the extinction probability of a single cell. For $\varrho = 0$, this is simply

$$\psi = \frac{\delta}{\delta + a}\frac{d+\beta+\alpha(\delta+a)}{\beta}, \tag{SI.39}$$

and hence the reconstitution probability given a dose of $\mathcal{S}$ donor HSCs is

$$\varphi = 1 - \left(\frac{\delta}{\delta + a}\frac{d+\beta+\alpha(\delta+a)}{\beta}\right)^{\mathcal{S}}. \tag{SI.40}$$
