# Supplementary figures and images for: Clonal dominance and transplantation dynamics in hematopoietic stem cell compartments
Source: PLoS Comput Biol. 2017 Oct 9;13(10):e1005803. doi: 10.1371/journal.pcbi.1005803 (PMC5654265; doi:10.1371/journal.pcbi.1005803)

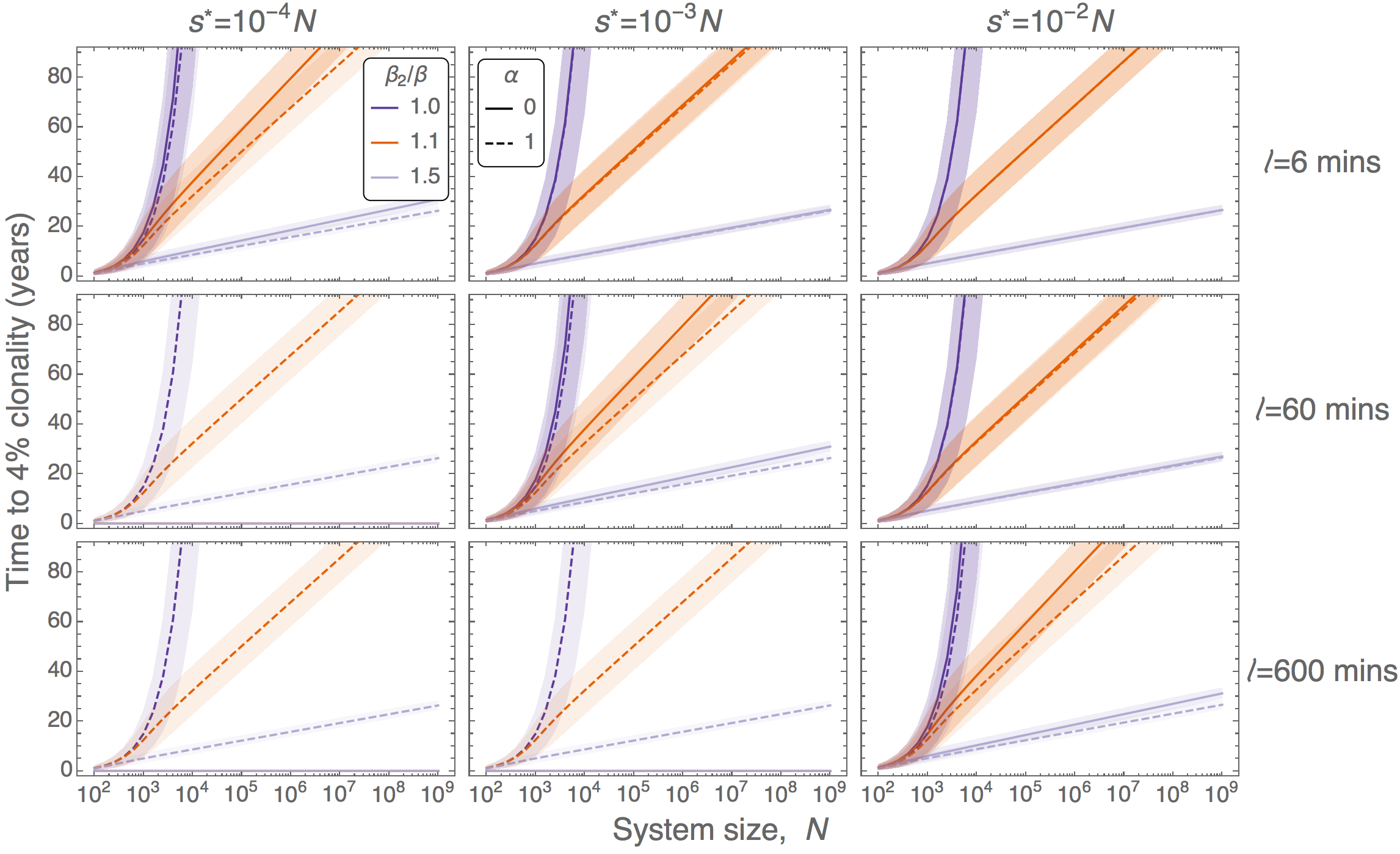

Supplement: S1 Fig — Time taken until a clone initiated from a single cell represents 4% [39, 40] of the human HSC pool, as a function of the total number of niches in the system. Colours represent the selective advantage of the invading clone. Solid lines correspond to death only within the niches (α = 0), while dashed lines represent equal death rates in both compartments (α = 1; see S1 Supporting Information for details). Lines are generated using mathematical formulae in the S1 Supporting Information. Remaining parameters are β = 1/40 week−1 [62], n* = 0.99N, and ϱ = 0. Some predictions are missing when d ≤ 0 and/or a ≤ 0; these parameter regimes are incompatible with our model. (TIFF) [file pcbi.1005803.s002.tiff]

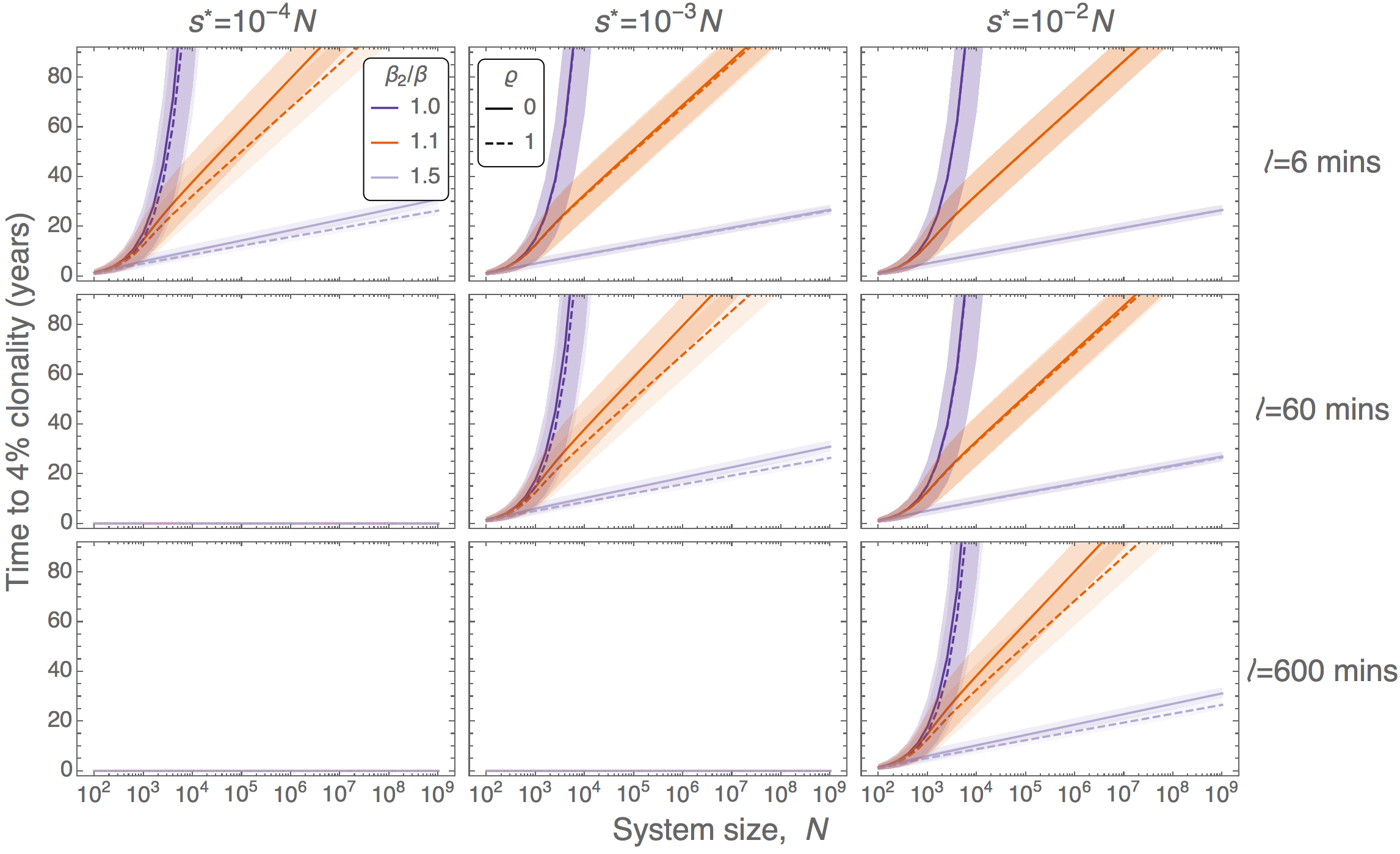

Supplement: S2 Fig — Time taken until a clone initiated from a single cell represents 4% [39, 40] of the human HSC pool, as a function of the total number of niches in the system. Colours represent the selective advantage of the invading clone. Solid lines correspond to the daughter cell entering the PB compartment after reproduction (ϱ = 0), while dashed lines represent daughter cells remaining in the BM (ϱ = 1; see S1 Supporting Information for details). Lines are generated using mathematical formulae in the S1 Supporting Information. Remaining parameters are β = 1/40 week−1 [62], n* = 0.99N, and α = 0. Some predictions are missing when d ≤ 0 and/or a ≤ 0; these parameter regimes are incompatible with our model. (TIFF) [file pcbi.1005803.s003.tiff]

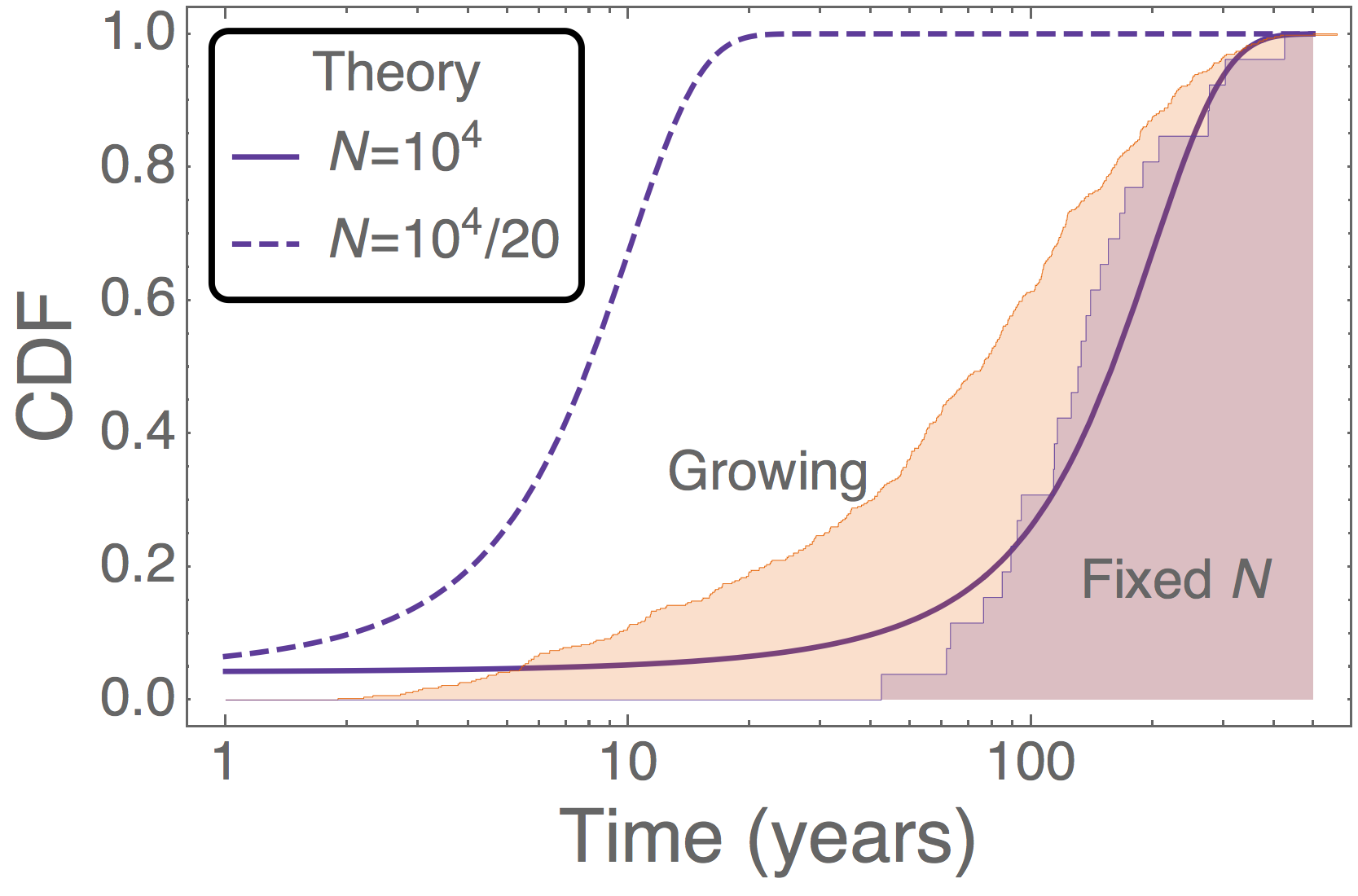

Supplement: S3 Fig — The cumulative probability density function (CDF) of times to reach 4% clonality [39, 40] when starting from a single neutral mutant in a normal host. Shaded regions are incidence curves from simulations using either a constant niche count of N = 104, or a logistically growing number of niches with N˙≈rN(1-N/K), where K = 104, r = 0.3 per year, and N(0) = K/20. These parameters represent a maturation period of ∼20 years to reach N ≈ K. Lines are predicted incidence curves which assume normally-distributed times to clonality, using the mean and variance formulae as described in the S1 Supporting Information, and constant population size as indicated in the legend (minimum and maximum number of niches). Remaining parameters are β = 1/40 week−1 [62], n* = 0.99N, s* = 0.01N, and ℓ = 60 minutes. Finally, we only consider here the conditional incidence time, which have been normalised by the fixation probability. This probability is 20 times larger for the neutral mutant in the growing model when compared to the fixed number of niches. (TIFF) [file pcbi.1005803.s004.tiff]
